# Improvement of diabetes-induced spinal cord axon injury with taurine via nerve growth factor-dependent Akt/mTOR pathway

**DOI:** 10.1007/s00726-024-03392-8

**Published:** 2024-04-18

**Authors:** Yachen Wang, Bihu Gao, Xiaochi Chen, Xiaoxia Shi, Shuangyue Li, Qing Zhang, Cong Zhang, Fengyuan Piao

**Affiliations:** 1 Stem Cell Clinical Research Center, The First Affiliated Hospital of Dalian Medical University, Dalian, 116011 China; 2 Department of Nephrology, Affiliated Zhongshan Hospital of Dalian University, Dalian, 116001 China; 3 Department of Urology, The First Affiliated Hospital of Dalian Medical University, Dalian, 116011 China; 4 Department of Occupational and Environmental Health, Dalian Medical University, Dalian, 116044 Liaoning China; 5 Department of Integrative Laboratory, Affiliated Zhongshan Hospital of Dalian University, Dalian, 116001 China; 6 Department of Nutrition and Food Safety, Dalian Medical University, Dalian, 116044 Liaoning China; 7 Department of Scientific Research Project, Affiliated Zhongshan Hospital of Dalian University, Dalian, 116001 China

**Keywords:** Diabetes mellitus, Diabetic neuropathy, Spinal cord axon injury, Taurine, Nerve growth factor

## Abstract

Diabetic neuropathy (DN) is a common neurological complication caused by diabetes mellitus (DM). Axonal degeneration is generally accepted to be the major pathological change in peripheral DN. Taurine has been evidenced to be neuroprotective in various aspects, but its effect on spinal cord axon injury (SCAI) in DN remains barely reported. This study showed that taurine significantly ameliorated axonal damage of spinal cord (SC), based on morphological and functional analyses, in a rat model of DN induced by streptozotocin (STZ). Taurine was also found to induce neurite outgrowth in cultured cerebral cortex neurons with high glucose exposure. Moreover, taurine up-regulated the expression of nerve growth factor (NGF) and neurite outgrowth relative protein GAP-43 in rat DN model and cultured cortical neurons/VSC4.1 cells. Besides, taurine increased the activating phosphorylation signals of TrkA, Akt, and mTOR. Mechanistically, the neuroprotection by taurine was related to the NGF–pAKT–mTOR axis, because either NGF-neutralizing antibody or Akt or mTOR inhibitors was found to attenuate its beneficial effects. Together, our results demonstrated that taurine promotes spinal cord axon repair in a model of SCAI in STZ-induced diabetic rats, mechanistically associating with the NGF-dependent activation of Akt/mTOR pathway.

**Supplementary Information:**

The online version contains supplementary material available at 10.1007/s00726-024-03392-8.

## Introduction

Diabetes mellitus (DM) is one of the most common chronic disorders. According to the statistics by International Diabetes Federation (IDF), there were 425 million individuals suffering from DM (18–99 years) worldwide, and the number was expected to reach 693 million by 2045 when DM should become the largest global epidemic in the twenty-first century (Ogurtsova et al. 2017). Along with the global increase of diabetic cases, the prevalence of its complications shall be expected to boost obviously.

As one of the most frequent complications of DM, diabetic neuropathy (DN) arises in nearly half of the patients (Dyck et al. 1993; Feldman et al. 2019). Rather than being a single entity, DM encompasses of several neuropathic presentations such as central nervous system (CNS) deficits as well as sensory and motor defects in peripheral nervous system (PNS) (Selvarajah et al. 2006; Fischer and Waxman 2010; Kou et al. 2014). DN is pathologically characteristic with axonal dysfunction, atrophy, and loss (Selvarajah et al. 2006). For example, postmortem studies showed axonal loss, gliosis, and demyelination in the spinal cord (SC) (Williamson 1904; Compston 2011). The involvement of SC in DN patients was evidenced even in early stage of DM according to a study by magnetic resonance imaging (Selvarajah et al. 2006). Such damage to SC was suggested inevitable (Boulton et al. 2005), and so far, no drug is available for neuronal loss. Even with the help of DM-control drugs, the development of DN can be postponed but cannot be completely prevented. Hence, it may be an important to look for strategies to promote neuronal regeneration and repair and functional restoration for DN patients.

As the most abundant free amino acid in human body, taurine has many crucial physiological functions, including osmotic regulation, neuromodulation, neurotransmitter regulation, and anti-oxidation (Sirdah 2015). Some recent studies have evaluated the therapeutic potential of taurine for DM, including some beneficial effects alleviating DM complications (Sirdah 2015). Taurine has been shown capable of improving hindlimb sciatic nerve motor function, digital sensory nerve conduction and blood supply and microcirculation of peripheral nerves in a study of Zucker diabetic rats (Li et al. 2006). Cubillos et al. reported that certain amino acids can accelerate neurite outgrowth of goldfish ganglion cells in retinal explants (Cubillos et al. 2000). Liu et al. suggested that neuronal regeneration was increased after maternal taurine supplementation in rat fetus suffering from DM-associated intrauterine growth restriction (Liu et al. 2015). Indeed, taurine concentration per se in SC has been found being elevated in response to spinal cord injury (SCI) in a rat model study (Diaz-Ruiz et al. 2007)*.* According to a recent study of SCI in lampreys, axonal regeneration was found to be promoted by taurine (Sobrido-Cameán et al. 2020). Taurine was also found to promote sciatic nerve repair in DM rats (Li et al. 2019a; Zhang et al. 2021). Nevertheless, the potential effect of taurine on spinal cord axon injury (SCAI) in DN, as well as its regulatory mechanism, has not yet been explored.

Because type 2 diabetes is the most common type of DM, we employed the streptozotocin (STZ)-induced rat DM model and studied the potential of taurine to alleviate SC-associated DN in this study (Zhang et al. 2021). In parallel, primary cultured neurons from rat cortex and VSC4.1 cells were used in vitro*,* exposing to high glucose (HG), in the absence or presence of taurine supplementation. Transmission electron microscopy (TEM) and immunofluorescent staining were used to track morphological changes of SC axons as well as neurite outgrowth of cultured cortical neurons after taurine supplementation. Behavior studies were used to evaluate sensory and motor function improvement. NGF expression and TrkA, Akt, mTOR phosphorylation were determined using western blot. In addition, NGF-neutralizing antibody, Akt and mTOR inhibitors were used accordingly. Our results showed that taurine promotes the repair of damaged axons in SC and improves neurological function recovery in DN. The NGF-dependent Akt/mTOR signaling pathway was found to be related to the beneficial effects of taurine. Our findings indicate that taurine could be used to ease the development of DM neurological complications.

## Materials and methods

### Animals

Animal experiments, including husbandry, handling, induction of DM symptoms and DN complications using streptozotocin, taurine treatment, neurological and behavior tests and necropsy procedure, were approved by the Institutional Animal Care and Use Committee of Dalian Medical University and followed the instructions of National Institute of Health Guide for Care and Use of Laboratory Animals. Sprague–Dawley (SD) rats aged 5 weeks were bred in Animal Experiment Center, Dalian Medical University. Animals were housed with standard conditions, including a 12-h light/dark cycle, room temperature (23–25 °C), free access to food and water.

### Antibodies

For western blot and immunofluorescence staining experiments, primary antibodies used were listed as following: growth-associated protein 43 (GAP43, Sigma-Aldrich, St Louis, MO, USA), Nerve growth factor (NGF, Cell Signaling Technology, Boston, MA, USA), TrkA receptor (TrkA, Cell Signaling Technology, Boston, MA, USA), Akt (Akt, Sigma-Aldrich, St Louis, MO, USA), Mammalian target of Rapamycin (mTOR, Abcam, Cambridge, MA, USA), pan-axonal neurofilament marker (SMI312, BioLegend, San Diego, CA, USA), myelin basic protein (MBP, Abcam, Cambridge, MA, USA), microtubule associated protein 2 (MAP2, Abcam, USA), DAPI (Abcam, USA) and β-actin (ACTB, ZSGB-BIO, Beijing, China).

### Experimental protocol

Sixty SD rats were randomly divided into five groups at (*n *= 12 for each group), as follows. (1) Control group: receiving routine diet and intraperitoneal (i.p.) injection with sham-control 1% citrate buffer. (2) DM group: receiving high-fat/sugar diet for 4 weeks. Afterwards, a single dose of i.p. injection of streptozotocin (STZ) (25 mg/kg, dissolved in 1% citrate buffer, pH 4.5). (3) (4) (5) DM + 0.5% Taurine (T) group, DM + 1.0% T group, and DM + 2.0% T group. Three days after STZ injection, they were randomly assigned into three treatment groups, and orally received double-distilled water containing taurine at doses of 0%, 0.5%, 1%, and 2% respectively. By blood collection from tail vein, fasting blood glucose levels were measured and used to evaluate successful establishment of type 2 diabetes in rats (3 days after intraperitoneal injection of STZ). Taurine administration was maintained for 8 weeks until necropsy. Spinal cord and cerebral cortex were collected.

### Transmission electron microscope

After being immersed in 2.5% glutaraldehyde and 0.1 M phosphate buffer (pH 7.4) at 4 °C for 6 h, spinal nerve samples (less than 1 mm^3^) were fixed in 1% osmium tetroxide for 2 h, followed by dehydration in gradient ethanol solution and entombment in naphthol. After ultra-thin (50–70 nm) tissue sectioning, slides were stained with uranium acetate and lead citrate and observed under transmission electron microscope (Carl Zeiss LEO 906E, Jena, Germany). Three sections per animal and four animals for each group were processed. Ten randomly selected fields were pictured per section, with particular focus on axonal diameter of myelinated fibers. Mean axonal diameter of rat spinal nerves were calculated using Image J (version 2) software.

### Function evaluation of sensory nerves

Thermal withdrawal latency (TWL), assessing the time interval in response to thermal stimulation, was measured by conducting plantar test after 8-week taurine or sham-control administration (Ugo Basile, Comerio Varese, Italy) (Cheng et al. 2021). Rats were placed individually on a glass plate, and after acclimatization, heat stimulation was applied with infrared intensity (IR) 50. The reaction time of rats was calculated as the time interval from the start-point of heat radiation to the start-point of paw withdrawal, being defined as TWL. The heat application was set up to be less than 20 s to avoid tissue damage. The TWL data were quantified by averaging three measurements with each interval being at least 10 min.

In addition, functional evaluation was also assessed using mechanical withdrawal threshold (MWT) as the time interval responding to mechanical stimulation. Each animal was placed in a plastic box and plantar surface of each hind paw was stimulated perpendicularly by employing a dynamic plantar aesthesiometer (DPA; Ugo Basile, Comerio Varese, Italy) (Cheng et al. 2021). Increasing force (2.5 g/s with a cutoff time of 20 s) was exerted using Von Frey-type 0.5 mm rigid filament until to paw movement. Quick withdrawal of hind paw under stimulation was deemed as positive response. For each animal, stimulation test was repeated by 3 times, with interval gap of 10 min. The average time length was calculated as PWT.

### Function evaluation of motor nerves

Rotarod test was conducted to determine motor function (Abdelkader et al. 2022). The rats were placed on a fixed rotarod and rested for 3 min to adapt before test. Then, the rod was rotated slowly at the beginning and the speed gradually increased with a maximal speed of 40 rpm within 5 min. The latency time from the test beginning until the rat falling off the bar was recorded. Each rat underwent three rotarod trials, with time interval being 5 min between each trial.

### Culture and treatment of ventral spinal cord 4.1 (VSC4.1) cells

VSC4.1 motor neuron, a fusion cell line of embryonic rat ventral SC neuron with mouse N18TG2 neuroblastoma cell, is a common tool used in SC neuron-associated studies in vitro (Wang et al. 2017). The cells were grown in monolayer, followed by sub-confluence in poly-L-ornithine-coated 75 cm^2^ flasks, with 10 mL of DMEM medium with 15 mM HEPES, pyridoxine, NaHCO3, 2% Sato's components, 1% penicillin/streptomycin (Beyotime, Shanghai, China), and 15% heat-inactivated fetal bovine serum (Hyclone, Logan, UT, USA). VSC4.1 cells were maintained in an incubator at 37 °C with 5% CO_2_. DMEM containing 2% FBS was applied to VSC4.1 cells for 24 h, followed by changing the cells to DMEM medium with glucose alone or with both glucose and taurine (10 mM, 20 mM and 40 mM). After treating for 48 h, it was needed to collect the cells and medium for next experiment.

### Primary culture and treatment of cerebral cortex neurons

Primary neurons harvested from neonatal cerebral cortex have been commonly used to assess neurite outgrowth of neurons in vitro (Jin et al. 2012; Zhou et al. 2014). Neonatal SD rats were sacrificed within 24 h after birth. Cerebral cortex was aseptically dissected and minced under a microscope, and then were transferred into a centrifuge tube containing L15 medium (Gibco, Gaithersburg, MD, USA). After wash with PBS, the L15 medium was added type II collagenase (St. Louis, MO, USA) and digested at 37 °C for 45 min. Later, after removing 2 ml of PBS with 20% fetal bovine serum, it was cultured at 37 °C for 10 min, and then, L15 medium with 1% BSA was used in washing, followed by inoculation at density of 5 × 10^5^ cells/mL. Subsequently, Ara-C with final concentration of 5 μg/mL was added after 24 h. Then, culture of cortical neurons was performed in DMEM/F12 for 48 h at 37 °C in a humid atmosphere with 5% CO_2_ and 95% air. After being seeded on poly-l-lysine-coated dishes with cell density of 5 × 10^4^/cm^2^ for 48 h in DMEM/F12 medium, cortical neurons was transferred to DMEM medium with glucose alone or with both glucose and taurine (10 mM, 20 mM and 40 mM). On the other hand, DMEM medium was treated with glucose alone or with both glucose and taurine (40 mM) containing NGF neutralizing antibody (10 μM), Perifosine (5 μM), and Rapamycin (20 μM) or not. After 48 h of treatment, the cells and medium were acquired for the later experiment.

For inhibitor or antagonist studies, cultured neurons were performed in medium with 150 mM glucose and 40 mM taurine, and pre-treatment was needed by employing NGF-neutralizing antibody (10 ng/mL, Abcam, USA), perifosine (5 μM, Beyotime, China), and Rapamycin (10 nM, Selleck, USA) or not. Cells underwent different treatments were cultured for 48 h at 37 °C.

### Western blot analyses of GAP-43, NGF, TrkA, Akt, and mTOR expression

Protein extracts were harvested from spinal nerve tissues of animal experiments and cultured cortical neurons or VSC4.1 cells using RIPA buffer (ThermoFisher). Protein concentration was determined using BCA assay. After electrophesis separation on SDS–polyacrylamide gel, proteins were transferred to PVDF membrane (Millipore, MA, USA), blocked using 5% nonfat dry milk in TBST (0.1% Tween-20). Primary antibodies, including GAP-43 (1:1000), MBP (1:1000), NGF (1:1000), Trk A (1:1000), Akt (1:1000), mTOR (1:1000), ACTB (1/4000), were used to incubate with membrane overnight at 4 °C. Horseradish peroxidase (HRP)-conjugated secondary antibody was used to incubate with membrane at room temperature for 2 h. Enhanced chemiluminescence reagent (Keyjen, China) was used to visualize the bands under the ChemiDoc-It Imaging System UVP Gel Imaging Analysis System. ImageJ was used to quantify the band intensity.

### Immunofluorescence staining of SMI312 and MBP

Spinal nerve or cortical neurons were fixed using 4% paraformaldehyde. Sections were incubated with blocking solution (containing 10% goat serum, 1% BSA, and 0.3% Triton 100 in PBS) for 1 h. SMI312 or MBP antibody was used to label axonal processes and primary antibody incubation was carried out overnight at 4 °C. Secondary antibody used species-specific fluorophore-conjugated immunoglobulin. DAPI was used to counter-stain nucleus.

### Morphometric analysis

Immunofluorescence images were obtained under microscope (Olympus, Tokyo, Japan) and analyzed using ImageJ (version 2) (National Institutes of Health, Bethesda, MD, United States). For morphometric analyses of spinal cord tissue sections, each treatment group included five animals, each animal’s tissue was processed into three independent tissue sections and each section was used to obtain ten fluorescent images. For morphometric analyses of coverslip-grown cultured cells, each treatment group included three coverslips and each coverslips were used to take ten fluorescent images and obtain ten fluorescent images. Fluorescent intensity was quantified as the integrated optical density (IOD) of per image in ImageJ. The neurite length of the cerebral cortex neurons was quantified by randomly selecting five fields per image and analyzed using ImageJ2. The number of neurites derived from neuronal cell bodies was also quantified (Zhou et al. 2014).

### Statistical analysis

Data were expressed as mean ± standard error (SEM), and data analyses were performed by Graphpad Prism 5 using one-way analysis of variance (ANOVA). Behavioral tests were analyzed using two-way ANOVA followed by Bonferroni post hoc analyses. Statistical significance was standardized as *p* < 0.05.

## Results

### Effect of taurine on SCAI in DN rats

**Fig.1:**
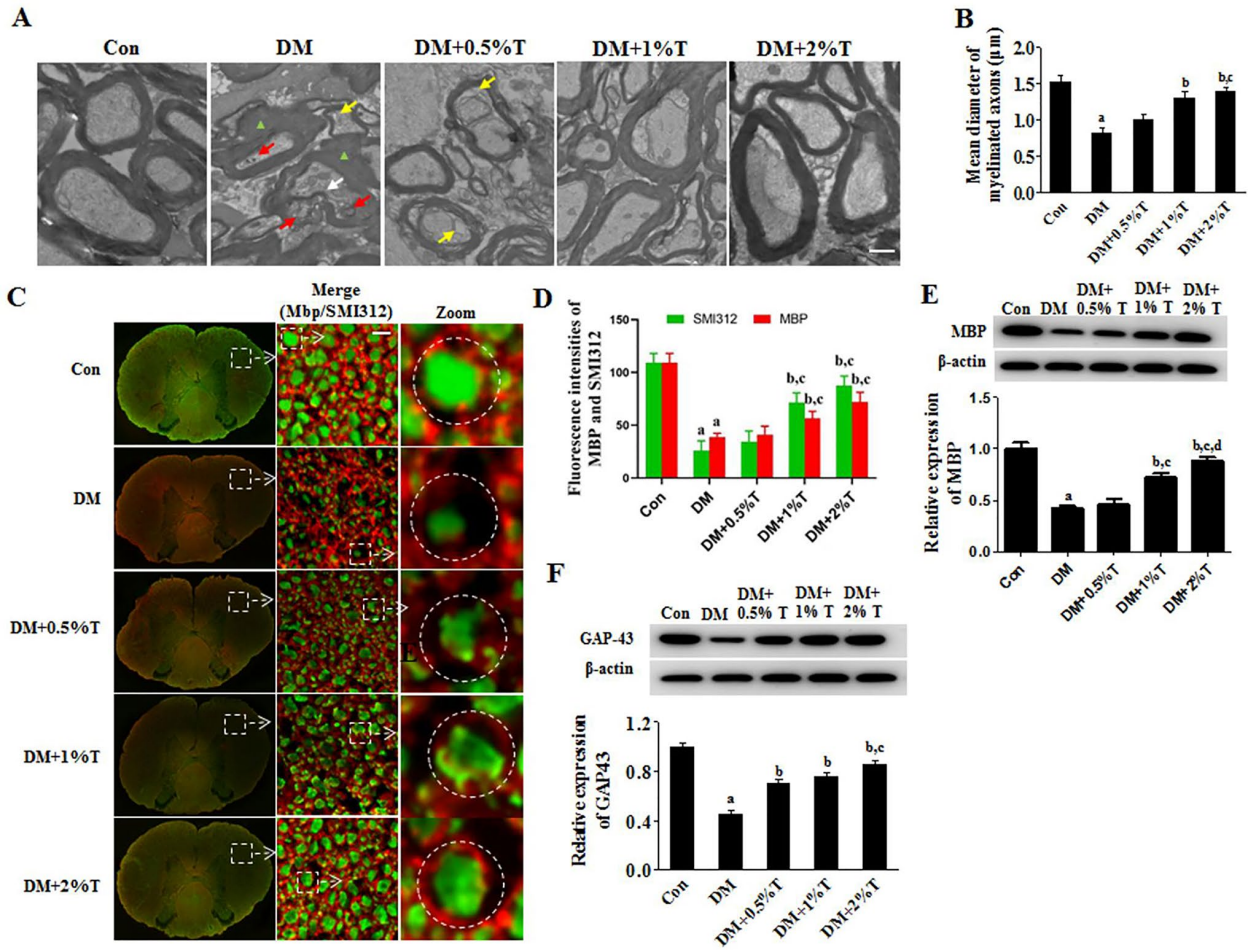
Effect of taurine on axonal morphology and GAP-43 expression in spinal nerve of DM rats. **A** Electron micrographs of morphological changes in SC of rat. Red arrows represented axonal regression, yellow arrows represented axonal vacuolization, green triangles represented irregular arrangement and disordered structure of the myelin sheath, and Bar was taken as 0.5 μm. **B** Mean diameter of myelinated axons in electron micrographs. Three sections were stained for each animal (*n* = 4), ten fields were randomly selected for each section, and observation was given to axonal diameter of myelinated fibers. Later, Fiji Image J2 software was used in calculating mean axonal diameter of spinal nerve of rats. **C** Double immunofluorescent images. As exhibited in the images, MBP (Red) and SMI312 (Green) were immunoreactive in transected spinal nerves. Bar was taken as 30 μm. **D** Fluorescence intensities of SMI312 and MBP in transected spinal nerves. Three individual sections were stained for each animal (*n* = 5), and ten fields were acquired in each section for morphometric analysis. **E** Effects of taurine on MBP expression of diabetic rats (*n* = 3). **F** Effects of taurine on GAP-43 expression of diabetic rats (*n* = 3). Data are expressed as mean ± SEM. ^a^*p* < 0.05 when comparing with Con group, ^b^*p* < 0.05 when comparing with DM group (DM), ^c^*p* < 0.05 when comparing with DM + 0.5%T group and ^d^*p* < 0.05 when comparing with DM + 1%T group

STZ-induced diabetic rodents are widely used in DN study (Metwally et al. 2018; El-Baz et al. 2020). In line with an earlier report that STZ-induced type 2 diabetic rats exhibited hyperglycemia, weight loss, elevated serum insulin, increased insulin resistance, and abnormal nerve conduction (Zhang et al. 2021), as shown in Fig. 1A, the axons of SC in our STZ diabetic rat model were characterized by morphological deformation, irregular arrangement, and pits and vacuoles under transmission electron microscopy. Moreover, comparing with control group, the mean diameter of myelinated axons in DN group was remarkably decreased (Fig. 1B). The myelin sheath presented with morphological changes with irregular arrangement and disordered structure.

Further evidence was found using immunofluorescence staining of pan-axonal neurofilament marker SMI312 and myelin basic protein MBP. In Fig. 1C, D, DM rats showed decreased fluorescence intensity of SMI312 or MBP staining in SC, comparing with control rats. Reduced protein expression level of MBP in was confirmed by Western blot (Fig. 1E). While GAP-43 expression level had relation with axon growth and motility, the diabetic rats of this study showed decreased expression of GAP-43 in SC tissue (Fig. 1F).

As shown in Fig. 1, comparing with the severe damage of SC in DN rats, Taurine treatment remarkably mitigated the abnormal morphological changes of axons, and enhanced the immunofluorescence intensities with SMI312 and MBP. Moreover, taurine increased GAP-43 and MBP expression in SC tissue. These results suggest that taurine promotes the regeneration of axons and attenuates the SCAI in DN. Taurine supplementation also eases myelin injury in SC of DN rats.

### Effect of taurine on nerve function recovery in diabetic rats

**Fig.2:**
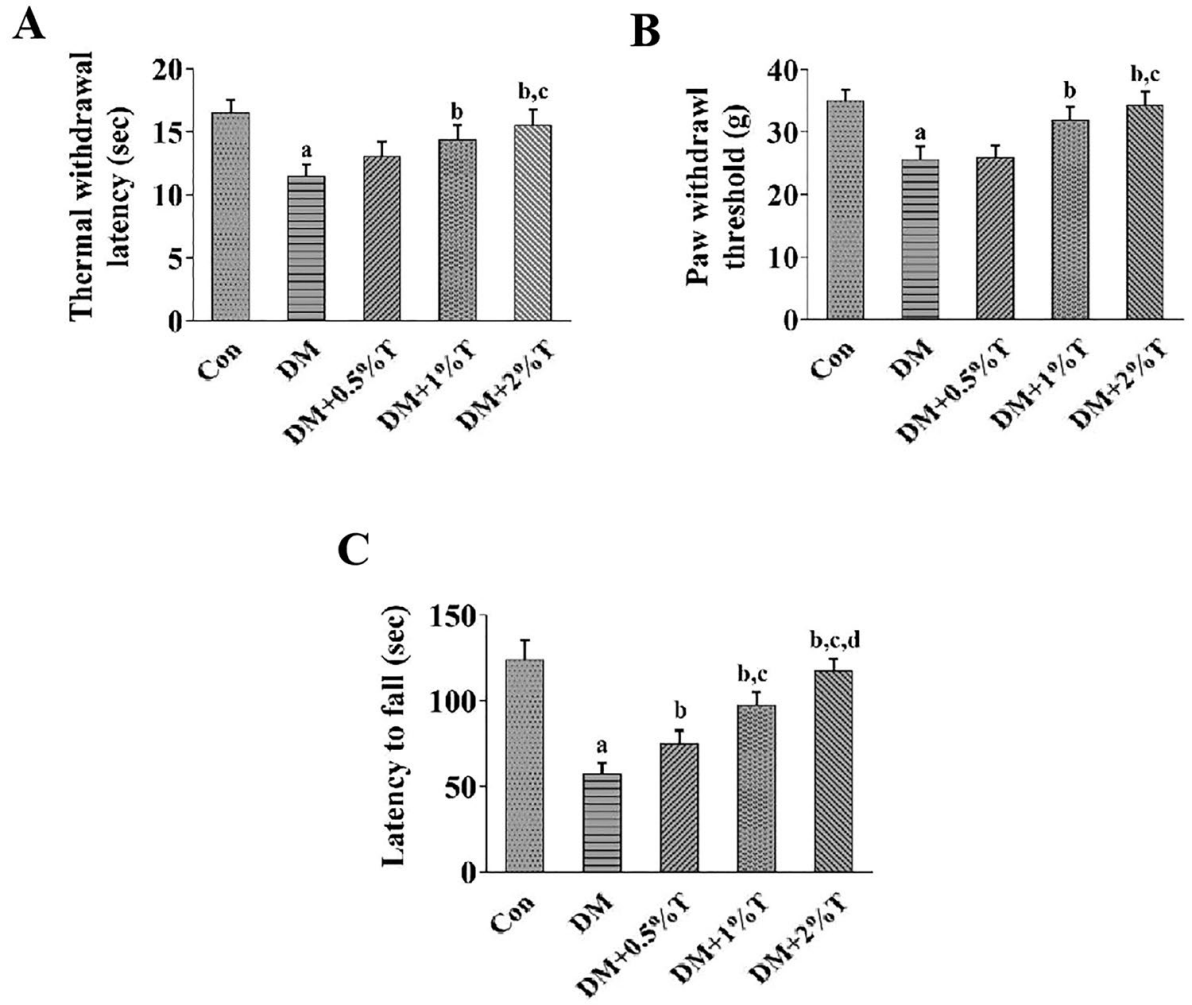
Effect of taurine on sensory and motor function of diabetic rats. **A** Effects of taurine on TWL of different groups (*n* = 10). **B** Effects of taurine on MWT of different groups (*n *= 10). At 8 weeks after taurine administration, TWL and MWT were adopted to evaluate sensory function and rotarod test was performed to check motor function as for each group of rats. This was repeated by 3 times as for each rat with time interval of 10 min, with the average taken as the TWL and MWT. **C** Effects of taurine on latency to fall of different groups (n = 10). Rotarod test used in evaluating motor function of the rats in each group at 8 weeks after taurine administration. By placing the rats on a rotating bar, the latency to fall was recorded. Each rat underwent 3 rotarod trials with time interval of 5 min. Data are expressed as mean ± SEM. ^a^*p* < 0.05 when comparing with Con group, ^b^*p* < 0.05 when comparing with DM group, ^c^*p* < 0.05 when comparing with DM+0.5% T group, and ^d^*p* < 0.05 when comparing with DM+1% T group

TWL and MWT were used to evaluate sensory function of rats in each group. According to Fig. 2A, B, TWL and MWT of diabetic rats were found to be decreased in comparison with the control group rats (*p* < 0.05). Nevertheless, taurine treatment greatly mitigated sensory nerve dysfunction in DN rats (*p* < 0.05). Rotarod test was conducted to evaluate motor function loss. The DN rats displayed a shorter latency to fall compared with the control group rats (*p* < 0.05), and such decrease of fall latency was increased markedly after taurine administration (*p* < 0.05) (Fig. 2C).

### Effect of taurine on neurite outgrowth of high glucose-treated cortical neurons

**Fig.3:**
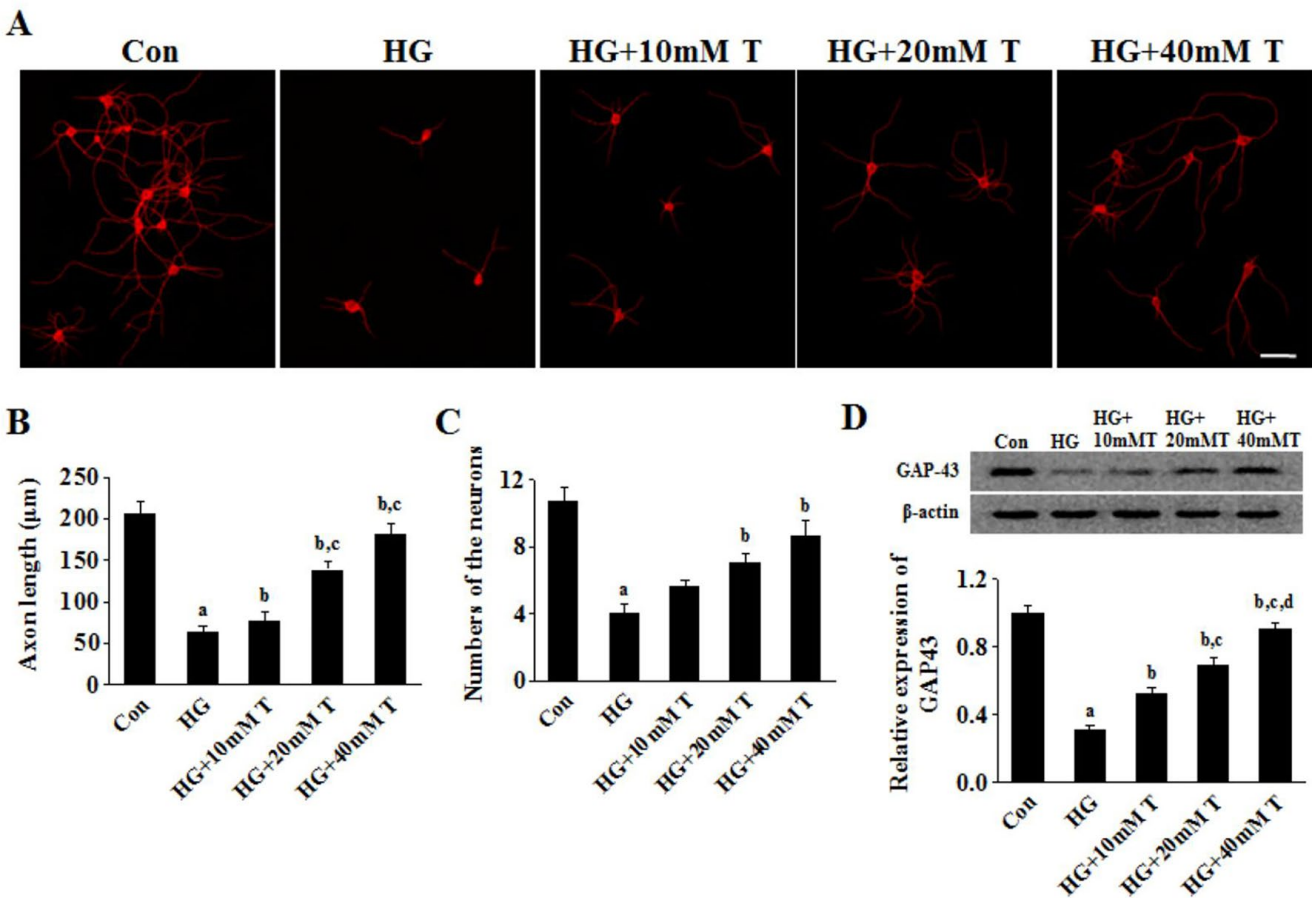
Effect of taurine on neurite outgrowth and GAP-43 expression in HG-treated cortical neurons. **A** Immunofluorescent images. As exhibited by the images, taurine promoted neurite outgrowth in HG-treated cortical neurons. Bar was taken as 50 μm. **B** Neurite length of cortical neurons. Three coverslips were stained for each treatment group (three experiment repeats), and ten fields were acquired in each coverslip for analysis. **C** Numbers of neurites. **D** Effect of taurine on GAP-43 expression in HG-treated cortical neurons. The effect was analyzed, and relative signal intensities of protein levels were shown against β-actin and quantified by employing Gel-Pro analyzer software. Data are expressed as mean ± SEM. ^a^*p* < 0.05 when comparing compared with Con group, ^b^*p* < 0.05 when comparing with HG group, ^c^*p* < 0.05 when comparing with HG + 10 mM T group, and ^d^*p* < 0.05 when comparing with HG + 20 mM T group

To further investigate the effect of taurine on neurite outgrowth, primary neurons were cultured with exposure of high glucose (HG). Fluorescent staining of SMI312 was used to assess neurite growth activity. As shown in Fig. 3A–C, neurite length of HG-treated cortical neurons was shorter comparing with that of control group neurons, and taurine administration increased the neurite length of cortical neurons in a dose-dependent manner. Further double immunofluorescent staining of the neurites using GAP43 and MAP2 antibodies also showed that the co-stained neurite of HG-exposed neurons was longer in the taurine group than the HG group (supplemental Fig. 1). Moreover, HG-treated cortical neurons exhibited decreased expression level of GAP43, and such decreased expression was up-regulated following taurine treatment (Fig. 3D).

### Effects of taurine on NGF expression and TrkA activation

**Fig.4:**
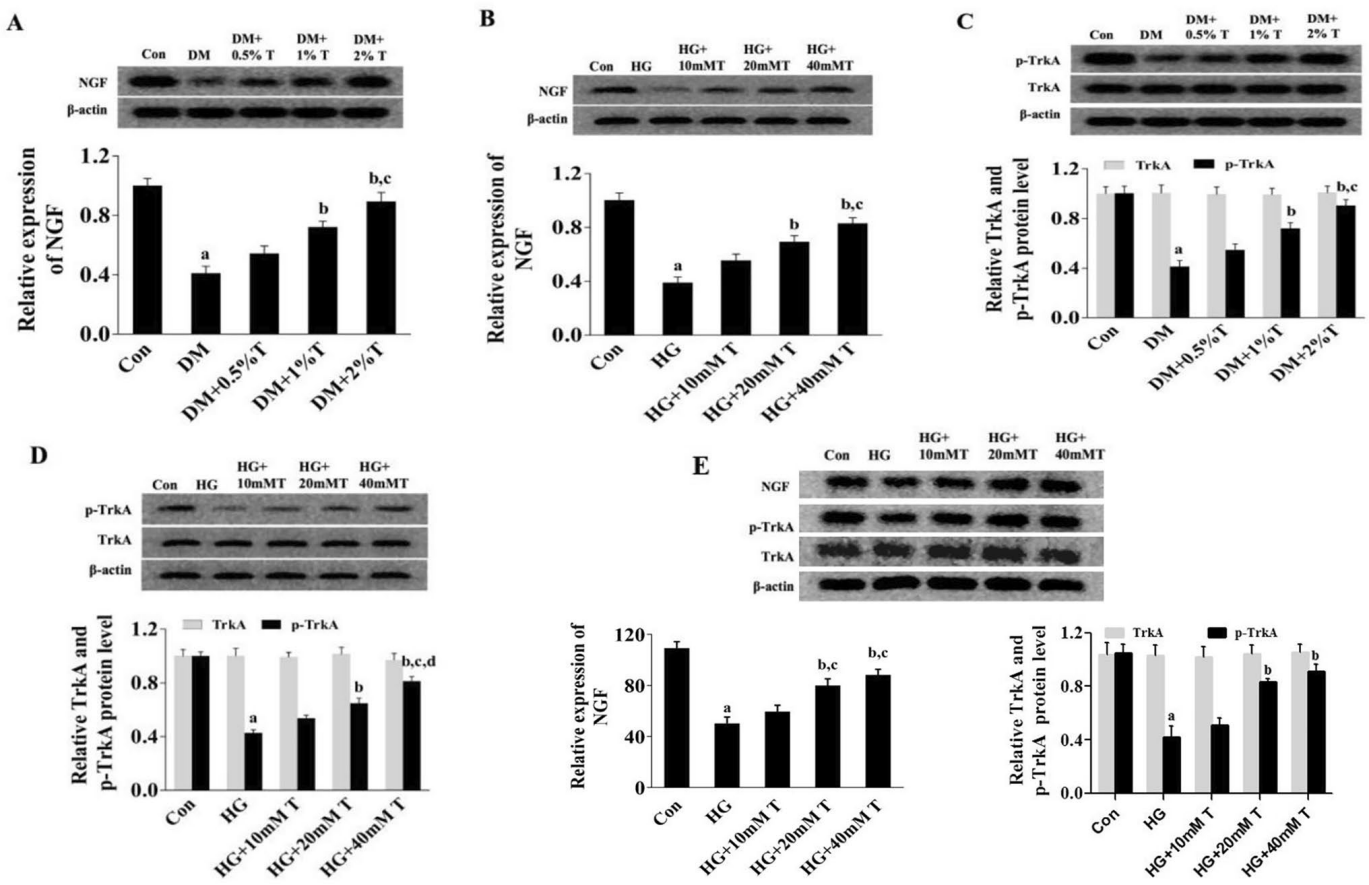
NGF and p-TrkA expressions in spinal nerve of diabetic rats and HG-treated cortical neurons in existence of taurine. **A** Effect of taurine on NGF expression in spinal nerve of DM rats. **B** Effect of taurine on NGF expression in HG-treated cortical neurons. **C** Effects of taurine on TrkA and p-TrkA expressions in spinal nerve of DM rats. **D** Effects of taurine on TrkA and p-TrkA expressions in HG-treated cortical neurons. **E** Effect of taurine on NGF, TrkA, and p-TrkA expressions in HG-treated VSC4.1 cells. Data are expressed as mean ± SEM (*n* = 3 for each group). ^a^*p* < 0.05 when comparing with Con group, ^b^*p* < 0.05 when comparing with DM or HG group, ^c^*p* < 0.05 when comparing with DM + 0.5% T or HG + 10 mM T group, and ^d^*p* < 0.05 when comparing with HG + 20 mM T group

As one of the essential neurotrophins, NGF plays an important role in neuronal growth and development. Recent literature suggested NGF deficiency in both animals and patients with DN that had close relation with neuronal death and impaired nerve repair (Wu et al. 2021). In the current study, NGF expression was determined in spinal nerves of DM rats or HG-treated cerebral cortex neurons/VSC4.1 cells with or without addition of taurine. As exhibited in Fig. 4A–E, NGF expression in SC of DM rats or HG-treated cerebral cortex neurons and VSC4.1 cells was obviously lower compared to that in the control groups (*p* < 0.05). The NGF expression level was found to be increased by taurine administration in a dose-dependent manner.

As the high affinity receptor-binding NGF, tyrosine kinase receptor type A (TrkA) triggers downstream signaling pathway with activating phosphorylation (Shintani and Noda 2008). In this study, phosphorylated TrkA level was down-regulated in spinal nerves of DN rats as well as cerebral cortex neurons/VSC4.1 cells with HG exposure comparing with control group (*p* < 0.05). Nevertheless, phosphorylated TrkA level was significantly up-regulated after taurine administration (*p* < 0.05) (Fig. 4C–E), indicating a potential effect elevating NGF expression and TrkA activation by taurine, which may be involved in the repair of SCAI in DN.

### Effect of taurine on Akt/mTOR pathway activation

**Fig.5:**
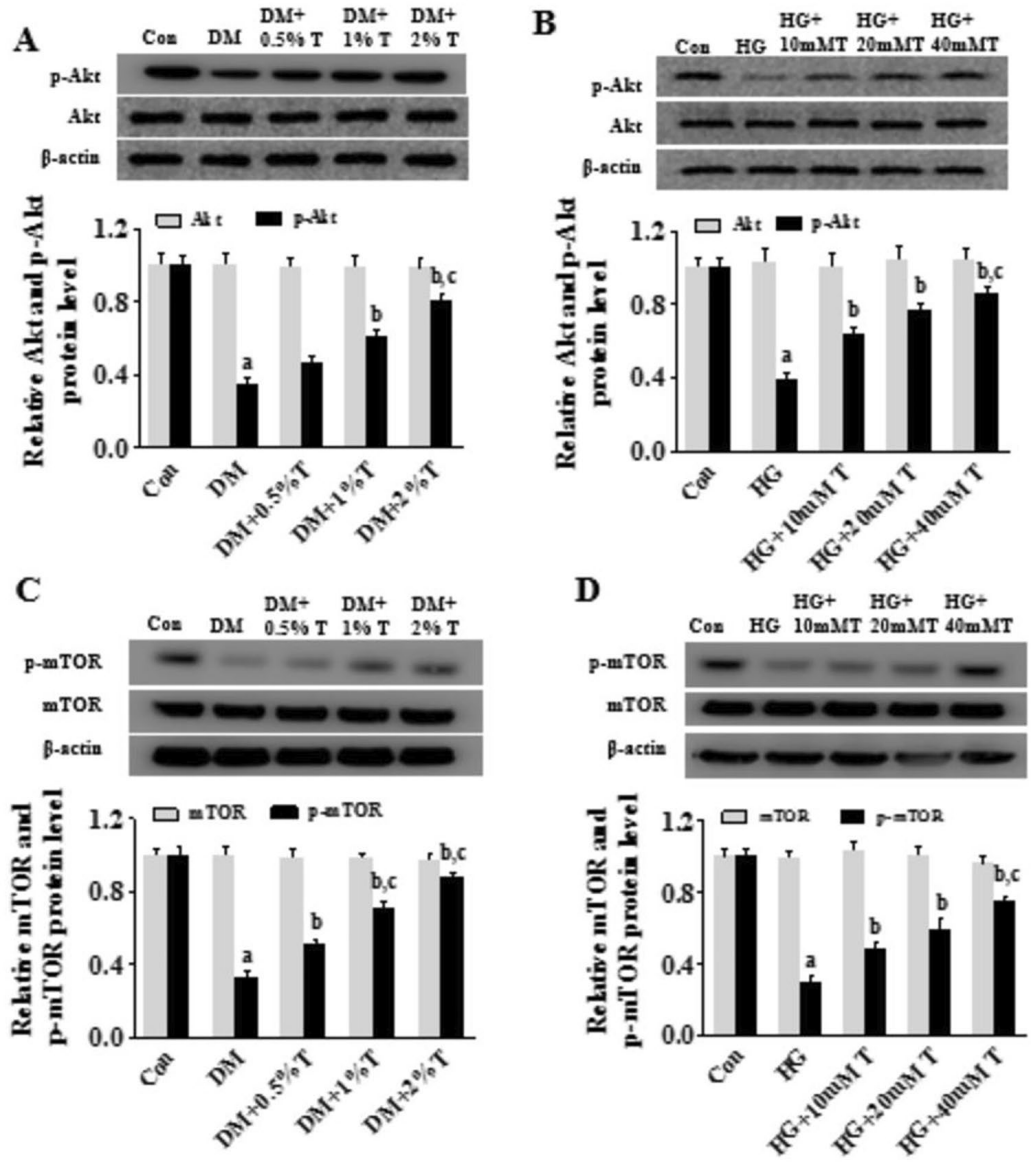
Akt, p-Akt, mTOR, and p-mTOR expressions in spinal nerve of DM rats and HG-treated cortical neurons in existence of taurine. **A** Effects of taurine on Akt and p-Akt expressions in spinal nerve of DM rats. **B** Effects of taurine on Akt and p-Akt expressions in HG-treated cortical neurons. **C** Effects of taurine on mTOR and p-mTOR expressions in spinal nerve of DM rats. **D** Effects of taurine on mTOR and p-mTOR expressions in HG-treated cortical neurons. Data are expressed as mean ± SEM (*n* = 3 for each group). ^a^*p* < 0.05 when comparing with Con group, ^b^*p* < 0.05 when comparing with DM or HG group, and ^c^*p* < 0.05 when comparing with DM + 0.5% T or HG + 10 mM T group

The binding of NGF to TrkA is known to activate phosphatidylinositol 3-kinase (PI3K)/serine/threonine kinase (Akt)/mammalian target of rapamycin (mTOR) pathway that mediates axon growth and nerve repair (Keeler et al. 2017; Sisti et al. 2021). To understand whether taurine treatment would activate PI3K/Akt/mTOR route to promote damage repair, we carried out experiment measuring the active phosphorylation signals of this pathway. pAkt level was found decreased (*p* < 0.05) in the spinal nerve of DM rats, as well as cerebral cortex neurons with HG exposure, whereas pAkt level was up-regulated notably after taurine administration (*p* < 0.05) (Fig. 5A, B). In addition, p-mTOR level was also downregulated (*p* < 0.05) in SC of DM rats and HG-exposed neurons and up-regulated (*p* < 0.05) after taurine treatment (Fig. 5C, D). These results suggested that taurine activates NGF/TrkA/PI3K/Akt/mTOR pathway in SC of DN.

### Indispensable role of NGF in taurine’s beneficial effect on nerve repair

**Fig.6:**
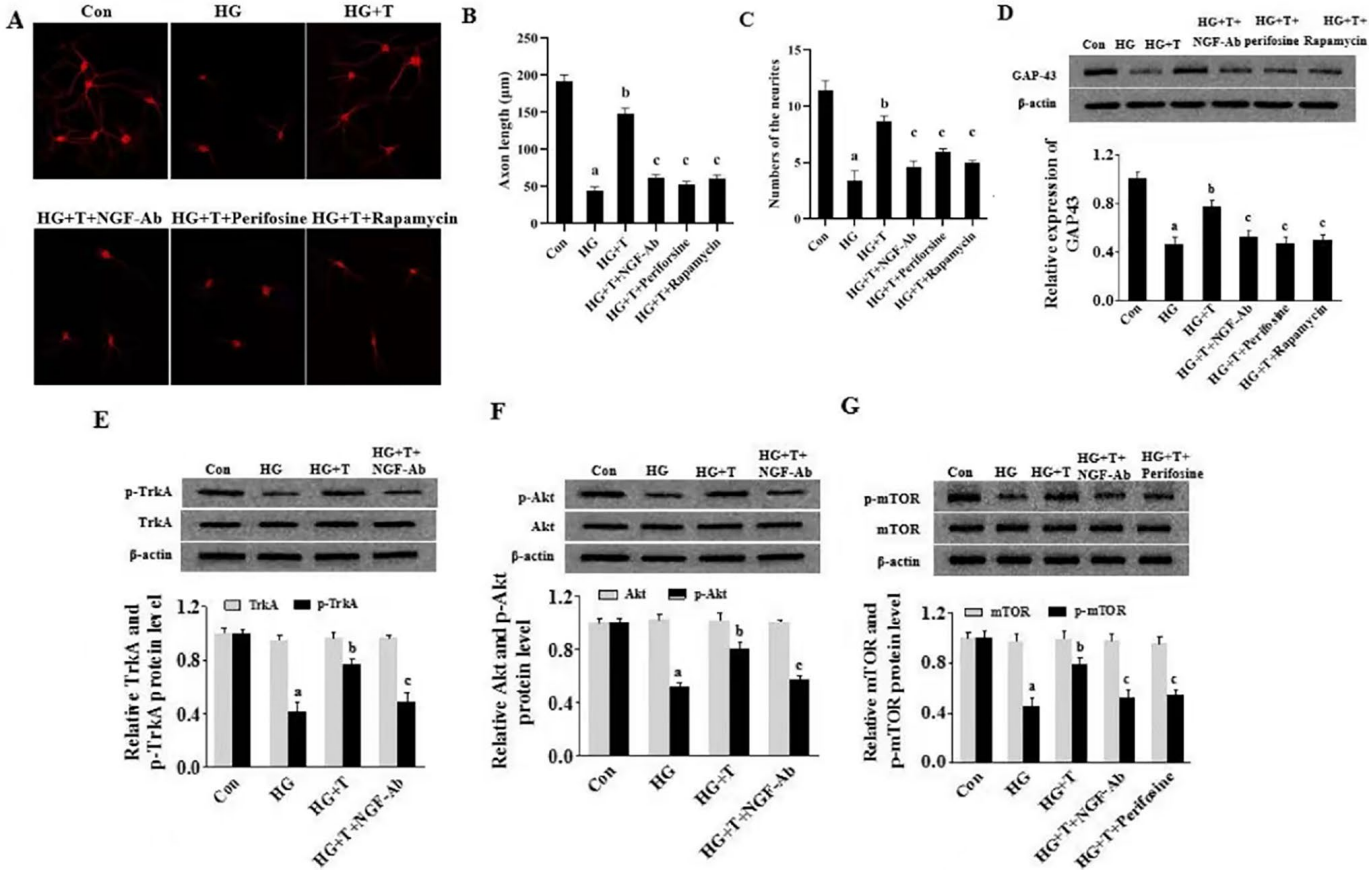
Promotion of neurite outgrowth in HG-treated cortical neurons by taurine via NGF-dependent Akt/mTOR signaling pathway. **A** Immunofluorescent images. As indicated by the images, effect of taurine on neurite outgrowth of HG-treated cortical neurons was blocked in existence of NGF antibody, perifosine, or rapamycin. **B** Neurite length of cortical neurons. **C** Numbers of neuritis. **D** Effect of taurine on GAP43 in HG-treated cortical neurons in existence of NGF antibody, perifosine, or rapamycin. **E** Effect of NGF antibody on TrkA and p-TrkA expressions in existence of taurine in HG-treated cortical neurons. **F** Effect of NGF antibody on Akt and p-Akt expressions in existence of taurine in HG-treated cortical neurons. **G** Effects of NGF antibody and perifosine on mTOR and p-mTOR expressions in existence of taurine in HG-treated cortical neurons. Data are expressed as mean ± SEM (*n* = 3 for each group). ^a^*p* < 0.05 when comparing with Con group, ^b^*p* < 0.05 when comparing with HG group, and ^c^*p* < 0.05 when comparing with HG + T group

To further demonstrate that the beneficial effect of taurine on axonal growth is dependent on NGF signaling, HD-exposed cortical neurons were exposed with various inhibitors together with taurine treatment. Interestingly, beneficial effects of taurine on neurite outgrowth and up-regulated GAP-43 expression were abolished after addition of NGF-neutralizing antibody (NGF Ab: ab16161), Akt inhibitor (Perifosine), or mTOR inhibitor (Rapamycin), respectively (Fig. 6A–D). In line with this, taurine treatment remarkably uprergulated phosphorylated levels of TrkA, Akt, and mTOR in HG-treated cortical neurons, whereas increased TrkA and Akt phosphorylation levels were blocked by NGF-neutralizing Ab, and the increased mTOR phosphorylation level was inhibited by NGF Ab or Perifosine (Fig. 6E–G).

## Discussion

In this study, we carried out experiments exploring taurine’s potential effect on diabetes-induced axonal injury of spinal nerve. In a summary, our results suggested that (1) taurine ameliorated SC damage in STZ-induced DM rats. (2) Taurine promoted axonal growth of SC, and recovered sensory and motor function. (3) Taurine increased NGF expression level that was lowered in SC of DN. (4) Taurine further activated the NGF-dependent TrkA/PI3K/Akt/mTOR pathway.

DN patients suffer from various motor dysfunction symptoms, such as gait problem, imbalance and body sway, ultimately increasing the risk of falling risk (Muramatsu et al. 2021). These motor system dysfunctions are associated with peripheral nerve damage as well as damages occurring in cerebral cortex and spinal cord (Ferris et al. 2020; Muramatsu 2020). Most studies of DN give considerable priority to PNS, with CNS being much less investigated. A pilot study reported that the pathogenesis of diabetic distal symmetrical polyneuropathy substantially involves damage of SC (Eaton et al. 2001). Another study confirmed the SC involvement in DN (Selvarajah et al. 2006). Upon exposure of glucose in high concentration, microvascular damage occurs body-widely, including SC tissue (Reske-Nielsen and Lundbaek 1968). Decreased conduction velocity and morphological abnormalities of sensory and motor axons were found in spinal cord of diabetic animal models (Muramatsu et al. 2018; Muramatsu 2020). In the present study, such damage were characterized by morphological deformation, irregular arrangement, pits and vacuoles. There was decreased SMI312, MBP and GAP-43 expression in SC tissue. Behavior tests showed decreased TWL and MWT index for sensory function loss, and latency-to-fall index for motor function damage.

The neuroprotective effect of taurine, a sulfur-containing amino acid that has been previously shown to participate in anti-oxidation, regulation of Ca2 + transport, osmoregulation, and anti-inflammation in various clinical and experimental studies (Schaffer et al. 2010), has been shown to ameliorate SC damage in our DN rat model. Indeed, taurine supplementation has shown in earlier studies to increase insulin sensitivity, normalize blood glucose level, down-regulate hyperinsulinemia, control hypertension, and control dyslipidemia (El Mesallamy et al. 2010). Furthermore, taurine supplementation has also been demonstrated to exert potential benefits for diabetic complications, such as retinopathy, nephropathy, neuropathy, atherosclerosis, and cardiomyopathy (Schaffer et al. 2010). Many investigators share particular interest in the therapeutic potential of taurine in diabetic neuropathy (Inam-U-Llah et al. 2018; Li et al. 2006; Rahmeier et al. 2016). In a study of the Zucker diabetic fatty rats, taurine was found to reverse deficits in hind limb sciatic motor and digital sensory nerve conduction velocity (Li et al. 2006). Our previous research also indicated that taurine could ease axonal damage in the sciatic nerve of diabetic rats and promote axon outgrowth in dorsal root ganglion (DRG) neurons exposed to high glucose. However, there are only a few reports investigating the beneficial effect of taurine in SCAI and its underlying mechanism. Sobrido et al. (2018) found that taurine promotes axonal regeneration after complete spinal cord injury in a lamprey model, being consistent with our results. Altogether, taurine may be a promising candidate for DN treatment.

Mechanistically, this study revealed that taurine’s beneficial effect is associated with its capability of promote NGF expression in neurological tissues. NGF plays an important role in regulating axon growth and guidance, resulting in neuroprotective and regenerative effects. Obrosova et al. reported that taurine could counteract NGF deficiency in diabetic peripheral neuropathy (Obrosova et al. 2001). Our previous studies suggested taurine supplementation exerts anti-apoptotic function via promoting NGF production in peripheral nerves (Li et al. 2019a; Wu et al. 2020). The present study found that taurine supplementation up-regulated NGF expression in spinal cord of DN rats, being consistent with earlier findings by us and others (Li et al. 2012, 2019b). The binding of NGF to its receptor TrkA is known to promote neuron survival, neurite outgrowth and axon regeneration driving by PI3K/Akt/mTOR signaling pathway (Yuan et al. 2003; Wang et al. 2016). Our in vitro and in vivo experiments showed that SC of DN rats and cultured neurons exposed to high glucose featured with decreased pTrkA, pAkt, and p-mTOR levels, whereas taurine treatment increased phosphorylation of these signal proteins. Using various inhibitors, we evidenced that the neuroprotection by taurine was largely dependent on the NGF/TrkA/Akt/mTOR signaling pathway.

The mechanism by which taurine up-regulates NGF expression in nervous tissue remains unclear. Several early reports suggested that oxidative stress is associated with decreased NGF concentration in neurological tissues of DM (Obrosova et al. 2001), whereas antioxidant therapy inhibited decrease of NGF (Garrett et al. 1997). It is possible that taurine possesses antioxidant properties to enhance cell survival and reduce apoptosis. However, in the present study we did not carry out further analyses in this regard. In our future study, to answer this question, inclusion of additional antioxidant treatment groups to our DN model and a comparison between the effect of taurine and the effect of antioxidants should be helpful. In addition, because streptozotocin is a potent generator of reactive oxygen species to damage many tissues and organs and taurine may exert antioxidant activities, the neuroprotective effect of taurine may partially originate from its ability to cleanse ROS.

Alternatively, the other neurotrophins, such as brain-derived neurotrophic factor (BDNF) and neurotrophin 3 (NT-3), also play vital roles in regulating growth, survival, and differentiation of neurons in CNS and PNS and, therefore, participate in nerve repair (Fang et al. 2017). Wu et al.’s study showed that taurine up-regulated BDNF expression in hippocampus in a rat model of depression (Wu et al. 2017). Therefore, it is not excludable that other neurotrophins may also involve in taurine’s neuroprotective effect. Hence, on-going experiments are carrying out to measure the concentration of other neurotrophins before and after taurine supplementation and to include additional BDNF and NT-3 neutralizing antibodies in taurine-treated neurons.

Finally, the present study used the STZ-induced type 2 diabete rat model to investigate taurine’s potential effect on neuropathy in spinal cord. Despite being a relatively old-fashioned animal model, various recent studies remain interested to use this model to gain some “rule of thumb” mechanistic understandings of diabetic neuropathy and its therapeutic strategies (Calcutt et al. 2017; Shen et al. 2024; Jin et al. 2024; El-Marasy et al. 2023). Besides, our findings through DN rat model was backed-up by our in vitro primary cortical neuron/VSC4.1 culture model, confirming taurine’s potential role of neuroprotection. As being discussed in one review by Islam MS and another by Kitada M et al. (Islam 2013; Kitada et al.^ 2016^), while many rodent models are available for diabetes research, the choice for studying diabetes neuropathy is relatively limited and more suitable for studying peripheral diabetic neuropathy. However, future experiments are in plan to investigate taurine’s protective effect on genetic induced rodent models, such as C57BL/Ks (db/db) mice model Genetically modified SDT fatty rat model (Yamaguchi et al. 2012; Hinder et al. 2013).

In summary, this study preliminarily suggested a protective role of taurine administration in ameliorating the damage to axons in SC of DN model and improving motor and sensory dysfunction. Mechanistically, taurine’s beneficial effect is likely through an NGF-dependent Akt/mTOR pathway. For its good safety proven in previous human clinical trials, taurine may be a promising candidate for nutritional or therapeutic supplementation for diabetic complications control.

## Supplementary Information

Below is the link to the electronic supplementary material.

Supplement Fig.1. Effect of taurine on neurite outgrowth and GAP-43 and MAP2 expression in HG-treated cortical neurons. (A)Immunofluorescent images. As exhibited by the images, taurine promoted neurite outgrowth in HG-treated cortical neurons. Bar was taken as 75 μm. (B) Amount of neurites per cell. Supplementary file1 (JPG 567 KB)
