# Supplementary figures and images for: Improvement of diabetes-induced spinal cord axon injury with taurine via nerve growth factor-dependent Akt/mTOR pathway
Source: Amino Acids. 2024 Apr 18;56(1):32. doi: 10.1007/s00726-024-03392-8 (PMC11026277; doi:10.1007/s00726-024-03392-8)

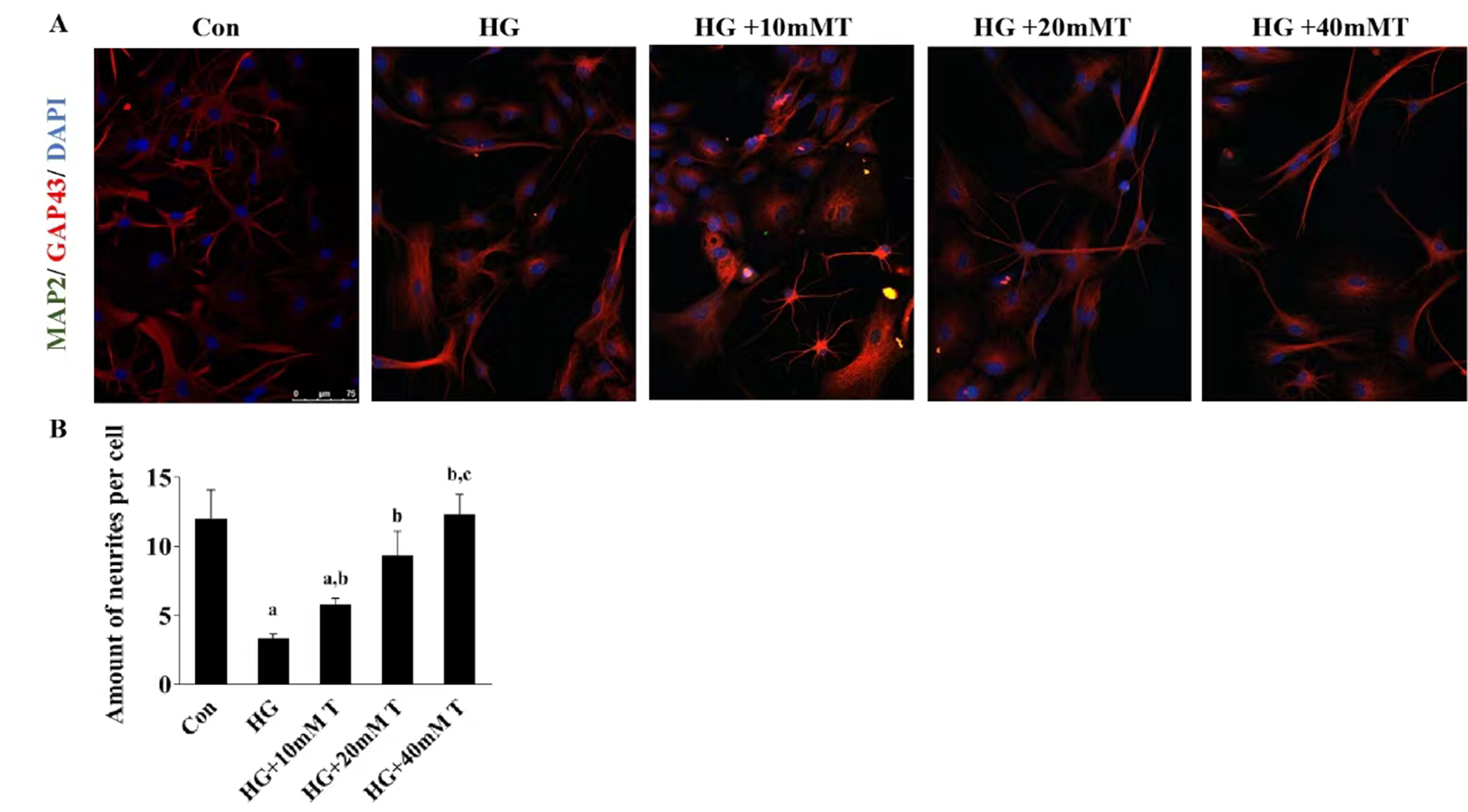

Supplement: Supplementary file 1 — Supplement Fig.1. Effect of taurine on neurite outgrowth and GAP-43 and MAP2 expression in HG-treated cortical neurons. (A)Immunofluorescent images. As exhibited by the images, taurine promoted neurite outgrowth in HG-treated cortical neurons. Bar was taken as 75 μm. (B) Amount of neurites per cell. Supplementary file1 (JPG 567 KB) [file 726_2024_3392_MOESM1_ESM.jpg]
